# Retraction: siRNA-mediated knockdown against NUF2 suppresses pancreatic cancer proliferation in vitro and in vivo

**DOI:** 10.1042/BSR-2014-0124_RET

**Published:** 2022-11-10

**Keywords:** NUF2, pancreatic cancer, RNA interference, treatment

This Retraction follows an Expression of Concern relating to this article previously published by Portland Press. This article is being retracted from *Bioscience Reports* at the request of the Editor-in-Chief and the Editorial Board following receipt of a notification from a reader alerting the Editorial Board to duplicated panels of figure 4c between this article and an article by Zhao et al (2016) (https://doi.org/10.1186/s40659-016-0086-3). The authors have been contacted with regards to the retraction but have not responded to the Journal's queries or the concerns raised. Given the extent of the issues raised, the Editorial Board stand by the decision to retract the article.

